# Stopping a Response When You Really Care about the Action: Considerations from a Clinical Perspective

**DOI:** 10.3390/brainsci11080979

**Published:** 2021-07-23

**Authors:** Sharon Morein-Zamir, Gideon Anholt

**Affiliations:** 1 School of Psychology and Sport Science, Anglia Ruskin University, East Road, Cambridge CB1 1PT, UK; 2 Department of Psychology, Ben-Gurion University of the Negev, Beer-Sheva 8410501, Israel; ganholt@bgu.co.il

**Keywords:** stop signal, response inhibition, proactive control, reactive control, Obsessive Compulsive Disorder

## Abstract

Response inhibition, whether reactive or proactive, is mostly investigated in a narrow cognitive framework. We argue that it be viewed within a broader frame than the action being inhibited, i.e., in the context of emotion and motivation of the individual at large. This is particularly important in the clinical domain, where the motivational strength of an action can be driven by threat avoidance or reward seeking. The cognitive response inhibition literature has focused on stopping reactively with responses in anticipation of clearly delineated external signals, or proactively in limited contexts, largely independent of clinical phenomena. Moreover, the focus has often been on stopping efficiency and its correlates rather than on inhibition failures. Currently, the cognitive and clinical perspectives are incommensurable. A broader context may explain the apparent paradox where individuals with disorders characterised by maladaptive action control have difficulty inhibiting their actions only in specific circumstances. Using Obsessive Compulsive Disorder as a case study, clinical theorising has focused largely on compulsions as failures of inhibition in relation to specific internal or external triggers. We propose that the concept of action tendencies may constitute a useful common denominator bridging research into motor, emotional, motivational, and contextual aspects of action control failure. The success of action control may depend on the interaction between the strength of action tendencies, the ability to withhold urges, and contextual factors.

## 1. Introduction

Effective stopping of maladaptive or inappropriate actions is closely linked to behavioural control and has long been of great interest to clinicians, cognitive psychologists, and neuroscientists. Inhibition of responses is assumed to contribute to adaptive, goal-directed behaviour. As such, it appears to be a useful construct with considerable face validity, often consisting of a clearly observable and intentional suppression of a planned, already initiated, or on-going action. Stopping of actions also appears in the conceptualisation of control processes such as executive functioning [1], and in this capacity could be representative of other self-regulation and control functions. It is also useful in clinically related theorising of numerous mental health disorders [2]. In fact, much of the literature focusing on cognitive neuroscience and experimental psychology makes reference to difficulties in response inhibition in various disorders as motivating the need for better understanding of the cognitive mechanisms, individual differences, neural systems, and neurochemistry involved. It is these difficulties in response inhibition that are apparent in mental health conditions such as Obsessive Compulsive Disorder (OCD). OCD has received considerable empirical and theoretical consideration in the response inhibition literature, making it a useful case study, and will largely be the focus of the present paper. Following a brief introduction to the broader construct of inhibitory control from a clinical perspective, we examine how response inhibition is explored empirically in controlled lab settings and the challenges and opportunities encountered in clinical research. We then consider response inhibition difficulties in the broader everyday context, where it is believed to underly maladaptive symptoms, and introduce a framework incorporating action tendencies and response inhibition with a goal of advancing the utility of response inhibition in clinical research. 

The importance of volitional behavioural and cognitive inhibitory control to clinical practice is apparent from inspection of the clinical criteria of mental health conditions. The DSM-5, one of the gold-standard diagnostic systems, outlines a list of criteria for each diagnosis, where patients typically must fulfil a minimum set out of a list of possible criteria over a given time period [3]. There are numerous DSM-5 criteria across a multitude of disorders that refer to instances of maladaptive behaviours where individuals appear to have difficulties in stopping, such as when struggling to overcome habits, to cease disruptive or maladaptive actions, or to resist temptations. For example, in OCD, where individuals experience unwanted obsessions and “attempt to ignore or suppress such thoughts, urges, or images”, typically unsuccessfully. Moreover, individuals with OCD are characterised by compulsions which are repetitive behaviours or mental acts an individual feels driven to perform and are clearly excessive, time-consuming, and often cause impairment in social, occupational, or other important areas of functioning. In Attention Deficit/Hyperactivity Disorder (ADHD), which is characterised by markedly different symptoms, diagnostic criteria include interrupting or intruding on others, blurting out answers, and ‘leaving their seat in situations when remaining seated is expected’. In the case of substance use disorders, stopping difficulties, again, appear key with individuals continuing to use, even when it causes problems, and wanting to cut down or stop using the substance but not managing to [3]. 

Taken together, from a clinical perspective, it is clear that whilst difficulties inhibiting responses are relevant to various disorders, the manifestations and instantiations in everyday life are varied and multifaceted. Moreover, whilst almost all individuals can exercise control and effective inhibition in some circumstances, it is the chronic, distressing, and impairing nature in everyday life attributable in part to behavioural disinhibition that characterise those who meet diagnostic criteria. Theorising of several disorders has consequently posited a central role for failures of cognitive control, and more specifically behavioural inhibition. In the case of OCD, inhibition difficulties have been proposed to underly many of the symptoms and neurocognitive findings [4]. Similarly, disruptions to inhibitory control in stimulant drug use are believed to contribute to various aspects of the disorder, including as a pre-existing vulnerability in at-risk individuals, through escalation to dependence, to promotion of relapse in chronic users [5]. The impaired response inhibition and salience attribution (iRISA) model specifically proposes that a decreased ability to inhibit maladaptive behaviours together with deficient salience attribution underlies addiction [6]. 

The presence and centrality of poor inhibitory control across distinct mental health conditions has contributed to its conceptualisation as a transdiagnostic construct [2]. That is, a core cognitive mechanism whose impairment contributes to the development or maintenance of multiple disorders. Indeed, response inhibition/suppression specifically appears under cognitive control in the Research Domain Criteria “matrix”, a framework advanced for integrating multiple levels of information and approaches to research mental disorders [7]. Not only that, but response inhibition specifically has been proposed as contributing to higher-order transdiagnostic constructs such as compulsivity and impulsivity, where in both self-restraint difficulties result in undesirable and maladaptive consequences [8]. Impulsivity encompasses actions and decisions that appear poorly conceived, prematurely expressed, and unduly risky [9]. Having received considerable theoretical attention, impulsivity has been fractionated into ‘motor impulsivity’ encompassing impaired response inhibition and ‘decisional impulsivity’, such as reduced delayed discounting of rewards [10]. Compulsivity on the other hand has been defined as repetitive acts, usually performed in the form of rigid rituals, accompanied by feelings of having to perform them while being aware that these acts are not in line with one’s overall goal [11,12]. Much of the overlap between these two independent and multifaceted constructs can be attributed to poor response inhibition and control, which likely contributes in distinct ways to the clinical presentation of each. For example, the persistence of inappropriate behaviours that result in negative consequences can be classed as impulsive when considering action initiation in the context of controlling desired or undesired urges but can also be classed as compulsive when relating to difficulties in stopping already initiated action or response sequences, leading to their repetitive nature. Further, response inhibition difficulties in and of themselves cannot be attributed to impulsivity or compulsivity necessarily, being associated with either. 

It is important to bear in mind that from a clinical perspective, difficulties in response inhibition are never considered to be the only cognitive impairment in a given disorder but operate as part of a range of general control-related issues (e.g., [13]). Inhibition typically encompasses not only response (or motor) inhibition but also cognitive inhibitory control, as measured by selective attention or conflict tasks such as Stroop, flanker, and Simon tasks. This is also consistent with research on executive functioning attributing inhibition to be part of a set of multiple yet separable general-purpose control mechanisms. One influential model posits inhibition within the context of cognitive flexibility or shifting and updating [1], though there is some uncertainty as to the exact and unique role of inhibition in this conceptualisation [14]. Indeed, in meta-analyses of cognitive functioning of OCD, case-control comparisons have comparable deficits of response inhibition to other executive function domains [15,16]. This finding is hardly unique to disorders of compulsivity and can also be seen in disorders of impulsivity such as ADHD [17,18]. 

## 2. Conceptualisation of Response Inhibition

### 2.1. Measuring Response Inhibition

Central to advances in the conceptualisation of response inhibition as described thus far has been the adoption of paradigms such as the stop signal task (SST) and go/no-go (GNG) task. Outside of clinical practice which relies on interview, observational, and self-report practices, these have allowed for individual-specific empirical measures of inhibiting prepotent responses in controlled settings. GNG tasks present go and no-go stimuli, requiring participants to respond to the former and withhold responding to the presentation of the latter, at times being viewed as response suppression. In contrast, stop signal tasks (SST) require participants to make a quick response to go stimuli presented on all trials but to countermand or inhibit these prepotent responses when stop signals are occasionally presented immediately after a go stimulus, thus being viewed as response cancellation [19]. The delay between the go and stop stimuli largely dictates whether a participant is likely to succeed in cancelling their planned response. The use of a fixed set of delays which is independent of participant performance involves a considerable number of trials [19]. In clinical research, the delay is often titrated for a given participant depending on performance using a staircase procedure, lengthening the delay duration following successful stopping and shortening it following failed stopping [20]. This enables performance to be assessed in a relatively brief period of time, thereby minimising testing duration and demands on participants. Nevertheless, the staircase procedure also introduces additional strategic demands as task parameters shift depending on participant performance. 

These inhibition tasks allow the measurement of response execution in the form of latencies to the go stimuli and their accuracy, but crucially also provide assessment of stopping performance, and as such are versatile in offering measures of multiple related outcome measures. In GNG, this is often in the form of commission errors. SST can also provide the proportion of stop trials where a participant failed to inhibit, but this is qualified by the specific delay values and the staircase procedure, which aims at achieving a success rate of approximately 50% to allow for estimation of stopping latencies. In any case, using the full distribution of go latencies alongside the rates of failed and successful stopping allows for the estimation of the time required to inhibit a response [19]. Although it cannot be observed directly, this stop latency is estimated by a race model, which in turn relies on several assumptions. The so-called stop signal reaction time (SSRT) is a key outcome measure of how quickly stopping is enacted and is often taken as a gauge of the efficiency of stopping and taken as the key stopping outcome measure [21]. 

Though the use of response inhibition tasks, and SST in particular, is flourishing in clinical research [21], there are discrepancies between the empirical measurement of response inhibition and its clinical conceptualisation. One key aspect is that both SSRT and commission errors measure stopping that is triggered in response to a clearly observable external stimulus (i.e., the stop or no-go stimuli, respectively). This responding to an external signal to stop can be considered a case of reactive inhibition. Reactive paradigms have been argued to be limited models of control as relevant to mental health disorders [22]. Endogenous signals or intent rather than external signals are an alternative prominent drive to stop or restrain one’s actions in everyday life that are of greater clinical relevance. Known as proactive stopping, this typically involves anticipating and preparing for the restraint of actions and adjusting behaviour accordingly [23]. Whilst clinical research into mental health disorders has predominantly focused on empirical assessment of reactive stopping, clinical presentation points to the importance of both reactive and proactive stopping as well as possible interactions between them (see below). Though far behind its reactive counterpart, proactive inhibition can be assessed in a complementary fashion. For example, in SST, it has long been known that compared to a block of go trials often presented as part of initial practice, the introduction of stop signals in a subsequent block leads to a general response slowing on go trials [24]. This has been attributed to participants anticipating the imminent need to stop and adjusting their overall response strategy proactively. Proactive stopping can also be investigated by changing the frequency of stop trials or manipulating the predictability that a stop will occur with the introduction of go- or stop-related cues [25,26,27,28,29]. Proactive stopping has also been considered in other paradigms, such as when stopping continuous actions or arm reaching, allowing for additional insight [27,30]. The explicit consideration of proactive inhibition in stopping tasks is the culmination of the recognition that multiple inhibitory control processes may be taking place in such tasks [23]. A challenge to clinical research is the adequate capture and identification of proactive versus reactive stopping given the potentially confounding roles of strategic demands across groups.

### 2.2. Response Inhibition Tasks in Clinical Research

There is considerable value in investigating stopping behaviourally from a clinical perspective. The stop-signal in particular holds appeal in this sense as it appears straightforward to administer to healthy individuals and patients alike in a relatively brief period of time. Moreover, task instantiation and administration may appear relatively uniform across studies, presumably yielding a comparable general marker of inhibition (i.e., SSRT). At the same time, the task can also be easily adapted to different purposes. However, it is important to consider the goal of any particular study. For example, studies commonly use cross-sectional design to establish whether a particular group, such as those with OCD, exhibit worse stopping performance as measured by SSRT compared to a control group, attributing this to differences in the effectiveness of suppressing responses. To date, such studies have focused largely on reactive stopping, noting medium effects, though against a background of considerable heterogeneity within groups and between studies [15,16]. These medium effect sizes indicate overlap between patients and controls, meaning there is a considerable subgroup of patients who have no discernible impairment. Employment of response inhibition tasks in clinical settings is not limited to cross-sectional studies. Stopping has been suggested as an endophenotype or biomarker offering insight into the underlying mechanisms of the disorder and with the possibility of being a potential predictor of therapy effectiveness [4,31]. Given its potential role in aetiology and maintenance, stopping is even being assessed as a target for intervention itself [32], though its suitability for uses aimed at capturing individual differences has been questioned [33,34]. 

As with all clinical research, any stopping impairments relating to a particular condition or their absence and the heterogeneity within patient groups must be interpreted in light of medication status and comorbidities [35,36,37]. Comorbidities in many mental health disorders such as OCD are the rule rather than the exception, though the most common conditions of anxiety and depression are not believed to be directly associated with response inhibition deficits [38,39]. Medication status may similarly contribute to heterogeneity in findings, with some evidence for differences in brain structure between medicated and medication-naïve OCD patients in areas associated with response inhibition [40]. Moreover, medication status may be confounded with pre-medication disorder severity, with those exhibiting more severe symptoms being more likely to be prescribed mediation, including dopaminergic drugs which are known to affect motor control and serotonergic drugs which affect some forms of motor restraint, though not SSRT [41]. Additional variation between studies may result from differences in task design and administration, which can have a considerable impact on performance and on the reliability and robustness of the outcome measures [21]. Heterogeneity in findings pertaining to SST in particular may result from the central role of task instructions and feedback [24], which govern the extent to which participants are likely to employ proactive inhibition. Experimenter bias may further play a role at times given the challenge for experimenters to be blinded to group status. Encouragingly, there is now clear consensus in the field as to SST administration and reporting practices [21].

A further challenge is that interpretation of findings from a clinical perspective is still far from straightforward. Even if there are robust response inhibition deficits in the majority or a proportion of patients, what may this mean? The presence of difficulties in stopping in cross-sectional correlational designs does not indicate a causal role in the development or maintenance of a disorder such as OCD [42]. For example, it has been proposed that inhibitory alongside other cognitive deficits in OCD result from acute and long-term effects of stress, as well as additional demands on attentional and control functions diverted to cope with or suppress obsessions and compulsions [15,43]. While assessing performance in first-degree biological relatives may speak to this, having indicated they too have some inhibitory deficits or abnormalities [31], it remains unclear exactly how such an underlying vulnerability directly contributes to symptom expression over time. Moreover, the relevance of stopping in particular for so many different and varied psychiatric conditions and seeming lack of specificity complicates its conceptual role. The neglect of proactive inhibition in cognitive theorising further contributes to the lack of conceptual clarity. No doubt the literature is set to explore proactive inhibition alongside reactive inhibition and the possible interactions between them, but many of the issues outlined above will still be relevant. Ultimately, greater clarity as to the purpose of investigating response inhibition in clinical contexts is also needed, be it to elucidate symptom-related mechanisms or in aid of diagnostic or intervention-related purposes.

### 2.3. On Caring about a Response: Internal and External Context

There is another underlying factor playing a role in the seeming discrepancies between the empirical response inhibition research and its clinical conceptualisation. On the one hand, clinical observations of OCD involve dramatic failures of control, with patients unable to refrain from performing rituals for up to several hours daily. This is not commensurable with the medium effect sizes reported above. A key issue that speaks to this is the relatively impoverished contexts in which countermanding and stopping are typically assessed [44]. This has already been acknowledged by many authors along with the recognition that most inhibitory actions be they reactive or proactive are shaped by memory and strategy [45]. Building on this, we argue that clinically relevant behaviours to an even greater degree are embedded in specific contexts and are shaped by affect and motivation driven by perceived rewards, or in the case of OCD, perceived threats. This is consistent with the appreciation that emotion and motivational processes influence and are often integral to cognitive control [46]. This also dovetails with the view that a comprehensive characterisation of executive functioning in general must encompass hot and cool cognition, as they are inextricably integrated within the human psychological experience throughout development and adulthood [47]. More specifically, individuals struggling with mental health conditions such as OCD will have difficulties suppressing maladaptive behaviours in specific circumstances, often in heightened states of arousal resulting from fear, anxiety, or negative urgency. For example, a patient who fears serious harm to their loved ones may find themselves unable to refrain from repetitively approaching and checking electrical sockets and cords but do this only in the home environment. Clinical observation indicates that a heightened state is not always needed as extensive, repetitive rituals can take place habitually with low affect and arousal. However, in these cases, individuals will typically report high distress if prevented or refrained from carrying out their compulsions or even at the mere thought of this happening.

One approach to address inhibitory control under such particular contexts is to introduce stimuli that are expected to elicit arousal or negative affect, as task-relevant or irrelevant. Numerous studies in non-patient samples have pointed to the deleterious effects of task-irrelevant negative emotion and arousal, presumably resulting from increased attention and processing of the stimuli [48,49,50,51]. Findings from task-relevant stimuli have been more mixed, for example emotive stop signals in the form of happy or fearful faces were found to lead to faster stopping compared to neutral faces, whilst a stimulus previously paired with a shock resulted in slower stopping [52]. Overall, research has pointed to complex interactions between inhibitory control, attention, and arousal, possibly explained by the inverted U function of arousal such that at times emotion can enhance performance whilst at others it can be deleterious. A similar experimental approach in OCD research builds on provocation studies [53] investigating inhibitory performance in response to symptom-specific stimuli. For example, by looking at inhibition to OC-related words [39]. This study found that compared to controls, patients with OCD were similarly impaired regardless of word content or the presence of comorbid depression, supporting a general inhibitory impairment. However, patients were even slower to switch responding away from the OC words, pointing again to the complex interplay between emotion (or arousal), attention, and executive control. General inhibitory impairments regardless of stimuli type are found in other conditions such as with addiction, whilst in others such as some eating disorders impairments appear in symptom-specific contexts [54,55].

A different approach explores how motivation may affect response inhibition by introducing incentives (e.g., monetary rewards) to standard stopping paradigms. In non-patients samples, it is evident that this can lead to complex strategic interactions, with participants adjusting responding to optimise performance given the modified task constraints [56]. Stopping can alternatively be enhanced, impaired, or seemingly unaffected depending on the aspects of performance that are being incentivised and the trade-offs between stop and go processes [56,57,58]. Though the introduction of rewards or punishments appear to trigger proactive control adjustments, their exact role is often unclear and complex [59]. The effects of motivation on inhibition in the form of rewards or punishments in OCD have not been extensively tested, in part due to concerns about confounding effects of arousal, anxiety, and mood. In a GNG study using punishment and reward, non-depressed patients with OCD did show reduced proactive control selectively under punishment conditions [60]. Monetary incentives are undoubtedly effective in changing the motivational state of participants and may point to important group differences. Moreover, failures of control in light of symptom-specific incentives or threats may offer additional insight, as suggested by evidence from addiction research [61]. This latter study also highlights that whilst response inhibition research often focuses on stopping efficiency, from a clinical perspective, it may be advantageous to investigate stopping failures and their triggers.

Theories of emotion typically recognise the interlinked nature of emotion, motivation, and action [62,63]. Action tendencies constitute a motivational component of emotion, focused on the preparation and direction of motor-related responses (e.g., approach or avoidance) [63]. Interestingly, using action tendencies as an important index of fear has been suggested as pivotal to advancing research in addressing the paradox between the adaptive nature of fear conditioning and the dysfunctional nature of pathological anxiety [64]. How proactive and reactive response inhibition may relate to action tendencies in such frameworks has yet to be explored. In OCD, as will be elaborated on later, action tendencies may be of particular utility due to the stereotyped, repetitive nature of compulsions. 

Taken together, the introduction of affective or motivationally relevant stimuli or contexts in response inhibition tasks takes steps towards greater clinical relevance, though often the interpretation of such findings can be more challenging. They also provide one strategy to bridging the gap between basic cognitive laboratory findings and everyday behaviour [34]. Careful consideration of the nature of stimulus content within and across groups can also offer insight into related cognitive processing in non-patient groups as OCD concerns such as contamination and infection are typically universal [65]. Moreover, a more holistic view of inhibitory control incorporating clearly defined and conventional operational measures of proactive control to complement those of reactive control should advance current understanding. Though no one study or indeed approach can sufficiently offer a complete picture, lab-based cognitive testing of reactive and proactive response inhibition can also provide systematic empirical and theoretical rigor. Moreover, they offer insight into hypothesised psychological mechanisms, capitalising on relatively low task variance between individuals [33]. This approach can thus help in ascertaining how any group differences may be related to abnormalities in general cognitive mechanisms or due to general distress, or alternatively whether they appear only in circumscribed situations or contexts with patient groups otherwise performing without impairments.

## 3. Clinical Theorising

### 3.1. The Cognitive-Behavioural Model of OCD

Taken together, basic cognitive approaches can offer insight into clinically relevant conditions but appear insufficient in advancing our understanding of the role response inhibition is playing in conditions such as OCD. Intriguingly, theorising and evidence from cognitive-behavioural appraisal accounts of OCD have focused on proactive rather than reactive control. According to such accounts, compulsive behaviours are triggered by normally occurring intrusive thoughts that are catastrophically interpreted as signifying harm and responsibility for its prevention [66]. Compulsive behaviours produce momentary relief, however they also provide negative reinforcement to symptoms and maintain dysfunctional interpretations [66]. For example, a patient with OCD may experience an intrusion “Did I leave the gas stove on?”. As a result, this patient may check and receive momentary relief, only to experience the same thought again (“Am I sure I turned off the gas?”), leading to an increased urge to check, thus getting entangled in a vicious cycle of doubt and checking. The repetitive thoughts and actions are not construed as failures in control, but as a paradoxical effect of suppression and compulsive behaviour [67,68]. 

Several beliefs have been suggested as etiological to the onset and maintenance of symptoms, or the motivation for proactive control. These include: (1) inflated responsibility, (2) over-importance of thoughts, (3) excessive concern about the importance of controlling one’s thoughts, (4) overestimation of threat, (5) intolerance of uncertainty, and (6) perfectionism [69]. However, multiple difficulties with this account were found, including absence of specificity to OCD and lack of these beliefs in a large percentage of individuals with OCD [70,71]. Initial evidence also does not seem to support a straightforward proactive control deficiency in OCD [72,73,74], though interestingly, one study examined this in paediatric OCD and did find decreased proactive control performance [75]. Therefore, there seems to be an impasse between cognitive suggestions of the deficits underlying symptoms and clinical accounts suggesting dysfunctional beliefs underlying symptoms, with both accounts thus far lacking.

In order to advance research, it may be useful to examine the seemingly key paradox that individuals with OCD present with: they desire to control and inhibit unwanted thoughts or urges but finding themselves being controlled by their recurrent compulsive behaviours and rituals. It may be the case that OCD patients demonstrate difficulties in the regulation of proactive control. Thus, they exhibit a general tendency of increased effort for proactive control, which is not relaxed even under circumstances where context suggests it beneficial to do so. There are various findings supporting this view. For example, in the Stroop task, OCD patients do not relax control even when neutral trial percentage is increased [76]. Another example of increased effort for proactive control in unwarranted circumstances concerns reduced Gratton effect for OCD patients on the Simon task [77]. A last example concerns an implicit learning task, where individuals with OCD demonstrated a preference for controlled rather than automatic processing [78]. Paradoxically, despite such seemingly greater efforts for proactive control, OCD patients and high OC participants were found to present with a reduced sense of agency, feeling as if “things happen” [79,80]. This decreased sense of agency may be construed as a sense of failure of reactive control. It is plausible to assume that increased efforts of proactive control and inevitable failures seen in reactive control [67] can result in a self-perpetuating vicious cycle. Investigating this cycle may enhance the understanding of OCD by assessing whether persistent and excessive increases in proactive control are associated with general or context-specific reduced reactive control. This line of reasoning points to the need to systematically investigate interactions between proactive and reactive control in conditions where they may fail. As with any useful account of OCD, contextual and state factors should help explain the reason that symptoms can occur in specific situations and with particular stimuli. For example, an individual with OCD may have difficulty resisting a strong urge to wash in response to experiencing contamination, whereas other important life domains remain unaffected. Hence, to examine the potential sources of control failure, we must also investigate the contextual factors that may influence emotional and motivational strength, or the difficulty to withhold a response in OCD. 

### 3.2. The Utility of Action Tendencies

Relatively little research has been conducted on assessing the strength of response in OCD, which may be more closely related to the phenomenology of the disorder in the context of failed inhibition. The construct of action tendencies may be useful in understanding increased urges to respond in OCD, in what is ultimately a maladaptive manner. According to Gibson’s affordances theory [81], when we perceive objects, not only do we perceive their physical properties, but also what we can do with them. Thus, various stimuli in particular contexts may be differential in their affordances, where stimuli associated with frequent responses involve strong action tendencies. Action tendencies may be understood not as a unidirectional consequence of emotion but rather bidirectional, where emotion-action tendency is part of a whole [82]. Obsessive thoughts in OCD can be viewed as action tendencies, which can either result from strong emotional states, or cause them. An example for the reverse directionality between action tendency and emotion is an experimental study with a student sample, in which participants were encouraged to increase checking of a daily performed activity, leading to increased threat perception of these activities (e.g., checking when locking the door, [83]). In OCD, certain internal or external stimuli are extremely potent in evoking symptoms. Such stimuli (e.g., a light switch, a gas knob) may be characterised by strong affordances, that may illicit increased action tendencies, experienced as urges, and potentially also failures in control. Compulsions may be the consequence of the activation of strong action tendencies, however at the same time may constantly perpetuate these action tendencies. Though additional research on the matter is needed, there is initial evidence for increased action tendencies in OCD [49,84]. The relation between action tendencies and inhibitory control must further be understood in light of both proactive and reactive control and their interactions. This may be particularly pertinent in OCD if there is potentially an overall tendency to prefer proactive control, which may increase action tendencies even for stimuli that are not subject to that control. According to this view, constant efforts of proactive control in OCD may (1) increase the strength of affordances of certain internal or external stimuli possess, and (2) increase susceptibility for reactive control failure. This points to the importance of action tendencies and specific emotions or motivations, together with interactions between reactive and proactive control as potentially underlying symptoms. 

If research supports this line of theorising, it may not only shed light on the underlying cognitive and psychological processes involved in OCD but also facilitate the development of more effective interventions. New interventions are needed because despite the success of existing first-line treatments, including exposure and response prevention and serotonergic medications, approximately half of patients do not show adequate response, with additional patients at a substantial risk of relapse [85]. Changing action tendencies as a clinical intervention has been incorporated in various psychotherapies, such as dialectical-behaviour therapy [86], and the unified protocol for the treatment of emotional disorders [62]. Moreover, the addition of fear antagonistic actions has been demonstrated to increase effectiveness of exposure treatment of acrophobia [87]. These antagonistic actions were in direct opposition to participants’ threat-relevant fear action tendencies (e.g., running towards the edge of a railing with eyes closed and hands behind one’s back). Of relevance, one study has found successfully reduced avoidance behaviour to contamination-related stimuli by modifying automatic action tendencies in an analogue sample with high contamination fears with the approach–avoidance task [88]. The addition of antagonistic actions to threat-relevant fear action tendencies in OCD may enhance treatment and adaptive control and could be explored by applying the response inhibition empirical and theoretical framework to actigraphy in the context of symptom provocation. Ultimately, interventions aimed at decreasing proactive control and increasing effective proactive inhibition in disorder-relevant contexts may complement other more general interventions aimed at improving restraint in everyday life [22]. 

## 4. Conclusions

In sum, we argue that to date, response inhibition paradigms such as the SST and GNG have been used to focus on reactive control, while proactive control failures may instead by more central in many disorders. Moreover, although research has often focused on group differences under impoverished contexts, clinical consideration suggests the need to further explore the specific circumstances under which patients experience failures of control, incorporating affective, motivational, and contextual factors. This alongside greater theoretical clarity as to what the role of any observed impairments may mean, will allow a more effective bridging between such lines of research and our understanding of symptoms as experienced in the everyday by patients. Though we present the arguments above with a focus on OCD, they are directly applicable to other mental health conditions, particularly those characterised by excessive impulsivity or compulsivity such as stimulant drug use. Further, there is also benefit in contrasting and comparing findings between disorders. We advocate for a close understanding of the issues faced by patients alongside careful appreciation of the cognitive theory and the strengths and limitations of different methodological approaches. Ultimately, given the complexity and richness of patient experiences, viable and useful advances will require a community of researchers and clinicians working together.
